# Effects of aquatic physical intervention on fall risk, working memory and hazard-perception as pedestrians in older people: a pilot trial

**DOI:** 10.1186/s12877-020-1477-4

**Published:** 2020-02-19

**Authors:** Michal Nissim, Abigail Livny, Caroline Barmatz, Galia Tsarfaty, Yitshal Berner, Yaron Sacher, Jonathan Giron, Navah Z. Ratzon

**Affiliations:** 1 Sackler Faculty of Medicine, School of Health Professions, Department of Occupational Therapy, Tel Aviv University, Tel Aviv, Israel; 2 Teachers for Students with Complex and Multiple Disabilities track, The David Yellin Academic College of Education, Jerusalem, Israel; 3 The Division of Diagnostic Imaging at the Sheba Medical Center, Tel-Hashomer, Israel; 4 The Sackler Faculty of Medicine, Tel Aviv University, Tel Aviv, Israel; 5 The Joseph Sagol Neuroscience Center, Sheba Medical Center, Tel-Hashomer, Israel; 6 Sheba Medical Center, Tel-Hashomer, Israel; 7 Sackler Faculty of Medicine, School of Health Professions, Department of Physiotherapy, Tel Aviv University, Tel Aviv, Israel; 8 IDC Herzliya, Herzliya, Israel

## Abstract

**Background:**

Normal aging is associated with balance, mobility and working memory decline that increase fall risk and influence activity of daily living functions. Mounting evidence suggests that physical activity is beneficial for decreasing aging effects. Previous studies have focused on land-based physical activity. Research concerning the aquatic environment is scarce.

The primary objectives of this three arm intervention pilot study were to examine the effects of an aquatic physical intervention program on balance, gait, fall risk and working memory among community-dwelling older individuals. The secondary objective was to examine the effects of an aquatic physical intervention program on safety of street–crossing among community-dwelling older individuals.

**Methods:**

Forty-two healthy participants aged 65 or older were enrolled into one of three intervention groups: aquatic physical intervention (API) (*N* = 13), on-land physical intervention (OLPI) (*N* = 14) or non-physical intervention (NPI) (*N* = 15). The intervention took place from 2018 until 2019 at Tel-Aviv University, Sheba medical center and Reich Center. The protocol included 30-min sessions twice a week for 12 weeks. Balance, gait and fall risk were assessed by the Tinneti test, working memory abilities were assessed by digit span and Corsi blocks tests and simulated safe streets-crossing was assessed by the hazard perception test for pedestrians.

Testing and data collection was conducted at baseline, after six weeks and 12 weeks of intervention. All members of the professional team involved in evaluating participants were blind to the intervention group to which participants were allocated.

**Results:**

The differences in Tinetti balance (F (2, 39)=10.03, *p* < 0.01), fall risk (F (2, 39)=5.62, p0 > .05), digit span forward (F (2, 39)=8.85, p < 0.01) and Corsi blocks forward (F (2, 39)=3.54, *p* < 0.05) and backward (F (2, 39)=6.50, p < 0.05) scores after 12 weeks between the groups were significant. The API group showed improved scores. The differences in hazard perception test for pedestrians scores after 12 weeks of intervention between the groups were marginally significant (F (2, 39)=3.13, *p* = 0.055). The API group showed improved scores.

**Conclusions:**

These findings may affect experts working with the elderly population when making decisions concerning therapeutic prevention interventions for the deficiencies of elderly patients. Older adults practicing aquatic physical activity could contribute to their increased safety.

**Trial registration:**

Trial registration number: ClinicalTrials.gov Registry NCT03510377. Date of registration: 10/31/2017.

## Background

Normal aging is associated with cognitive decline [1] such as diminished working memory (WM) [2–5], and impairment in motor performance [6] such as reduced balance and mobility [7]. These deteriorations in cognitive and motor performance may influence activity of daily living functions, [8] such as safe street crossing. WM is a complex cognitive function that enables goal-directed behavior. It has a limited capacity for storage, update and manipulation of content [9]. WM is important for making appropriate road-crossing decisions, such as selecting a safe time to step into oncoming traffic. Balance and mobility decline may lead to an increased risk of falling and fall-related injuries [10–12]. Reduced mobility and balance may lead to slower walking speed, imparing the ability to cross streets safely due to longer exposure time to traffic [13, 14]. According to the National Highway Traffic Safety Administration in the United States, about 34% of pedestrians injured and about 4% killed in road crashes were elderly individuals [15]. Furthermore, up to 50% of all injured pedestrians in Organisation for Economic Co-operation and Development countries are elderly individuals [16]. In line with these statistics and the rising worldwide phenomenon of the growing number of elderly individuals in the general population [17, 18], it is important to find an effective intervention method to improve the safety of elderly people.

Various forms of on-land physical interventions have been found beneficial to promoting WM [19–24],balance and mobility [25, 26]. As the environment of the physical activity is important to the outcomes of the activity, changing the environment to an aquatic setting may increase the benefits of the intervention [27]. An individual immersed in water is exposed to physical forces (specific gravity, thermodynamic and the meta-centric effects) different from those on land due to the density and viscosity of water [28]. Immersion improves balancing abilities by increasing the proprioceptive input on the immersed body. Promoting body awareness increases sensory feedback, as resistance to movement through water is greater than resistance to movement through air [28]. Therefore, immersion in an aquatic setting provides multi-sensory stimulation, combining three sensory systems: the vestibular, proprioceptive, and tactile, which may help improve balance and coordination [29, 30]. Yet, there are few studies examining the effects of Aquatic Physical Intervention (API) on cognitive abilities [31–34] and functional behaviors [35, 36].

The present study proposed a physical intervention method using Tai-Chi and Ai-Chi techniques. Tai-Chi has been used as an effective way of improving motor and cognitive abilities [37, 38]. Tai-Chi was originally developed as a form of martial art in China, but has been practiced as a physical exercise, mainly by elderly population, because of its low speed [39]. The Ai-Chi method is based on Qigong and Tai-Chi movements [40]. Previous studies found improved positive effects of both Ai-Chi and Tai-Chi on static and dynamic equilibrium, fall risk [41], and positive effects of Ai-Chi on verbal WM ability [32].

Based on the aforementioned studies, the primary objectives of the present study were: 1.To examine the effects of API on balance, mobility, and fall risk compared to identical on-land physical intervention (OLPI) and cognitive non-physical intervention (NPI) in the elderly population2. To examine the effects of API on verbal and visuospatial WM compared to identical OLPI and cognitive NPI in the elderly population. The secondary objective was to examine the effects of API on simulated hazard-perception as pedestrians to identical OLPI and cognitive NPI in the elderly population.

## Methods

### Design and participants

**Fig. 1:**
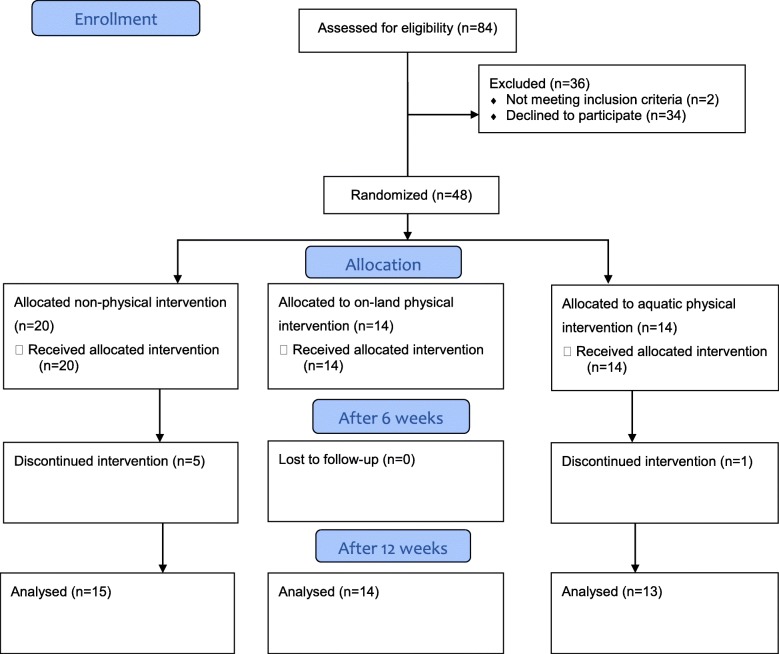
The consort flow diagram

The current study was a three-arm pilot trial. A total of 42 adults aged 65–89 years (M = 74.4 ± 6.65) participated in this study. The study population was recruited from elderly day care centres though ads and social media (Facebook). All participants were independent and consented to the study by signing a consent for provided by a research assistant. To exclude potential effects of depression and cognitive impairments, participants had to score below 10 on the Geriatric Depression Scale [42] and above 24 in the Mini Mental State Examination [43]. Other exclusion criteria included: 1. a medical history of neurological, orthopedic or psychiatric conditions causing permanent impairments, 2. the use of drugs that may cause dizziness in accordance to the guidelines of the manufacturer, and 3. an absence from intervention exceeding two weeks. All participants who met the inclusion criteria were randomly allocated to three intervention groups: 13 participants attended the structured API, 14 participants the structured OLPI, and 15 participants the NPI (Fig. 1). Simple randomization was generated and obtained by a research coordinator with three exceptions: 1. the exclusion of two couples (husband and wife) who were randomly allocated to different intervention groups but asked to be in the same group or drop out from the research. 2. One man who was allocated to the aquatic intervention, thatasked to be placed in a group located close to his home and was therefore allocated to the structured OLPI group. 3. Five subjects dropped out of the study from the NPI group, leading to the recruitment of five additional subjects from the same day care centers by identical ads, into the NPI group. As a result, 16.66% of participants were not randomly allocated. Future studies should consider a larger study population at the beginning of the study in order to have a better randomization process.

The sample was estimated based on digit span forward test values found previously [32], with 80% power and a standard deviation of 0.63, which can be achieved with a minimum of *n* = 10 subjects in a group. The sample size provided a minimally acceptable probability of incorrectly failing to reject the null hypothesis that there is no difference between the groups. Therefore, the projected minimum cohort was 10 participants per group in order to ensure a medium effect size.

#### Interventions

The intervention protocol took place from 2018 until 2019 at Tel-Aviv University, Sheba medical center and Reich Center, and included a 30-min exercise session conducted twice a week for 12 weeks, for a total of 24 sessions. Four instructors conducted the intervention. All instructors were certified hydrotherapist Ai-Chi instructors or Tai-Chi instructors, and were trained for the intervention protocol to ensure identical intervention in all groups. In addition, a research coordinator followed the research protocol once a week.

#### API

The Ai-Chi method, based on Qigong and Tai-Chi movements, was selected for the structured API [40]. For the present study, 16 movements were used from the Ai-Chi method. The first six movements were more static and symmetrical while the other movements were focused on continuously changing the center of gravity and center of buoyancy. The Ai-Chi intervention was conducted in a hydrotherapy pool (34 °C) approved by the Ministry of Health.

#### OLPI

For the controlled comparison of the structured on-land motor intervention, 16 identical movements were used in the Ai-Chi method.

#### NPI

Participants in the NPI group practiced guided imagery of the 16 identical movements used in the Ai-Chi method (listening to the instructor’s voice) while sitting on a chair.

Both the OLPI and the NPI were conducted in a quiet room. Primary outcomes measures:

The Tinetti Balance and Gait test [44]: a standardized evaluation of balance and mobility designed to determine risk of falls in the elderly. All items in both balance and mobility sub-tests are scored (0–2). Tinetti scores for risk of falls are: ≤18 points = high risk; 19–23 points = moderate risk; ≥24 points = low risk.
(a) Digit span test forward (DSF) and (b) Digit span test backward (DSB) [45]: a verbal WM test using digit recall [46]. During the task, a sequence of digits is read by the experimenter and participants are asked to recall the digits in forward or backward order immediately after hearing them. The task starts with a sequence of two digits, and the number of digits per sequence is increased by one if a participant successfully recalls a given sequence length twice. Performance is rated by the number of sequences successfully recalled.
Corsi block-tapping test forward (CBTF) and backward (CBTB) [47] test of visuospatial WM. During the task, the participant watches the tester touch a series of blocks, then is asked to touch the blocks in the same order or backward. The task starts with one block, and the number of blocks per sequence is increased by one if the participant successfully recalls a given sequence length twice. Performance is rated by the number of sequences successfully recalled.

Secondary outcomes measures:

4. Hazard-perception test for pedestrians (HPTP) [48]: is a computerized instrument that was designed to efficiently test and train pedestrians with regard to safe crossing and detection of road hazards. A video clip with traffic scenarios, including various approaching hazards, is shown on a screen. Hazard detection (potential hit) is identified by pressing the spacebar on the keyboard whenever a potential hazard is detected. A detection time-frame is calculated for each potential hazard in each clip, which lasts from the moment the potential hazard appears on screen until the moment it would hit the pedestrian. Pressing the spacebar during the detection time frame signals to the HPTP software that a potential hazard is detected. Each detection time frame is divided into five equal segments, and the detection score depends on the segment during which the spacebar is pressed. Scores for each potential hazard detection range from 5 (highest) to 0 (lowest), so that pressing the spacebar during the first detection time-frame segment receives a full score [5], during the second segment 80% of the full score [4], and so on. Pressing the spacebar before or after the detection time frame produces no score (0). The final score for each clip is calculated as the sum of scores divided by the number of potential hazards in that clip. The test consists of 10 clips for each scenario. The HPTP software presents the clips in random order

Testing was conducted at baseline, after six weeks and after 12 weeks of intervention by two qualified occupational therapists and one qualified learning disability teacher,trained to perform the test protocol. All members of the professional team involved in evaluating participants were blind to the intervention group to which participants were allocated.

### Statistical analyses

All analyses were performed using IBM-SPSS v.23 (IBM-SPSS Statistics for Windows, Armonk, NY: IBM Corp., USA). A two-sided *p*-value≤0.05 was considered statistically significant. A chi-square test of goodness-of-fit was performed to determine goodness of fit of bio-demographic parameters between the three intervention groups. A One-way ANOVA analysis was conducted to ascertain that the participants did not differ in the experimental parameters measured between groups at the beginning of the intervention.

To test the effect of each intervention program on the experimental parameters measured in the current experiment, relative change between time measurements in each parameter was calculated. The effect of the intervention programs was calculated by extracting the differences in scores between the baseline measurement and six or 12 weeks of intervention in each parameter. To test whether the difference in scores between time periods varied between intervention groups, a One-way ANOVA analysis was conducted following with Tukey post hoc test.

Data was also calculated according to the intention to treat analysis guidelines by reducing seven participants (16.6%) from the main sample (one from the API group, one from OLPI group and five from the NPI group that were not recruited randomly). This analysis did not yield signtificant changes in the results that were obtained when analysing the whole sample of the experiment. Therefore, the results that are displayed further represent the previous analysis.

## Results

There were no statistical differences between intervention groups in all bio-demographic variables (Table 1) and all measured parameters at base-line (Table 2(. All experimental parameters measured between groups after 12 weeks of intervention are presented on Table 3. All experimental parameters measured between groups after six weeks of intervention are presented on Table 4.

**Table 1:** Participants’ demographic variables across intervention groups

		n	OLPI	n	API	n	NPI
		14		13		15	
Gender							
Male			3 (25%)		4 (33%)		5 (41%)
Female			11 (91%)		9 (75%)		10 (83%)
Family status							
Single			2				
Married			7		10		5
Widower			3		2		5
Divorce			2		1		4
Other							1

**Table 2:** Experimental parameters measured between groups at the beginning

	n	OLPI mean (SE)	95% Confidence interval	n	API mean (SE)	95% Confidence interval	n	NPI mean (SE)	95% Confidence interval	Sig.
	14			13			15			
Tinetti balance (0–16)		13.6(.53)	12.80—14.47		13.4(.55)	12.45—14.46		13.9 (51)	12.47—15.39	n.s
Tinetti gait (0–12)		11.5(.31)	10.95—12.04		11.5(.32)	10.81—12.26		11.6(.299)	10.88–12.31	n.s
Digit span test forward (0–16)		8.7 (.53)	7.64–9.92		8(.55)	7.11–8.88		9.6(.513)	8.29—10.90	n.s
Digit span test backward (0–14)		6.0(.47)	5.06—6.93		5.5(.49)	4.34—5.96		6.6(.46)	5.36—7.83	n.s
Corsi block-tapping test forward (0–16)		6.7(.45)	5.85—7.57		7(.475)	6.25—7.74		6.6(.442)	5.45—7.88	n.s
Corsi block-tapping test backward (0–14)		6.42(.40)	5.53—7.32		7(.42)	6.25—7.74		6.2(.39)	5.25—7.14	n.s
Hazard perception test for pedestrians (0–5)		1.56(.18)	1.13—1.99		1.38(.18)	1.07—1.69		1.93(.17)	1.52—2.34	n.s

**Table 3:** Experimental parameters measured between groups after 12 weeks of intervention

	n	API mean (SD)	n	OLPI mean (SD)	n	NPI mean (SD)	F	*P* Value
	14		13		15			
Tinetti balance (0–16)		1.385(.53)		0.357 (1)		0.133 (0.91)	10.03	P < 0.001
Tinetti gait (0–12)		0.23(.77)		0.14(.77)		0.20(.77)	0.52	n.s
Fall risk		1.69 (1.03		0.50 (1.45)		0.06 (1.38)	5.62	*P* < 0.0.5
Digit span test forward (0–16)		0.214 (.80)		1.84 (1.06)		0.26 (1.34)	8.85	P < 0.001
Digit span test backward (0–14)		0.92 (1.11)		-0.07 (1.2)		0.33 (2.02)	1.437	n.s
Corsi block-tapping test forward (0–16)		1.07 (1.03)		0.00 (1.17)		0.66 (1.27)	3.54	P < 0.0.5
Corsi block-tapping test backward (0–14)		0.92 (0.86)		0.14 (1.09)		−0.47 (1.06)	6.50	*P* < 0.0.5
Hazard perception test for pedestrians (0–5)		0.39(.50)		0.19(.59)		0.32 (0.50)	3.13	P = 0.055

**Table 4:** Experimental parameters measured between groups after 6 weeks of intervention

	n	API mean (SD)	n	OLPI mean (SD)	n	NPI mean (SD)	F	P Value
	14		13		15			
Tinetti balance (0–16)		0.846(.80)		0.071 (1)		0.66(.74)	6.00	P < 0.0.5
Tinetti gait (0–12)		0.23(.59)		−0.14(.36)		0.20(.77)	0.52	n.s
Fall risk		1.07 (1.25)		0.26 (1.09)		−0.07(.82)	4.06	P < 0.0.5
Digit span test forward (0–16)		1.23 (.92)		0.07(.99)		−0.33 (1.54)	6.20	P < 0.0.5
Digit span test backward (0–14)		0.769 (1.16)		0.21 (1.05)		0.00 (1.0)	1.882	n.s
Corsi block-tapping test forward (0–16)		0.84(.68)		−0.14 (2.2)		0.06 (1.70)	1.28	n.s
Corsi block-tapping test backward (0–14)		0.46)0.77)		−0.57 (1.45)		0.53 (1.24)	3.19	*P* = 0.0.52

### Primary outcomes measures

**Fig. 2:**
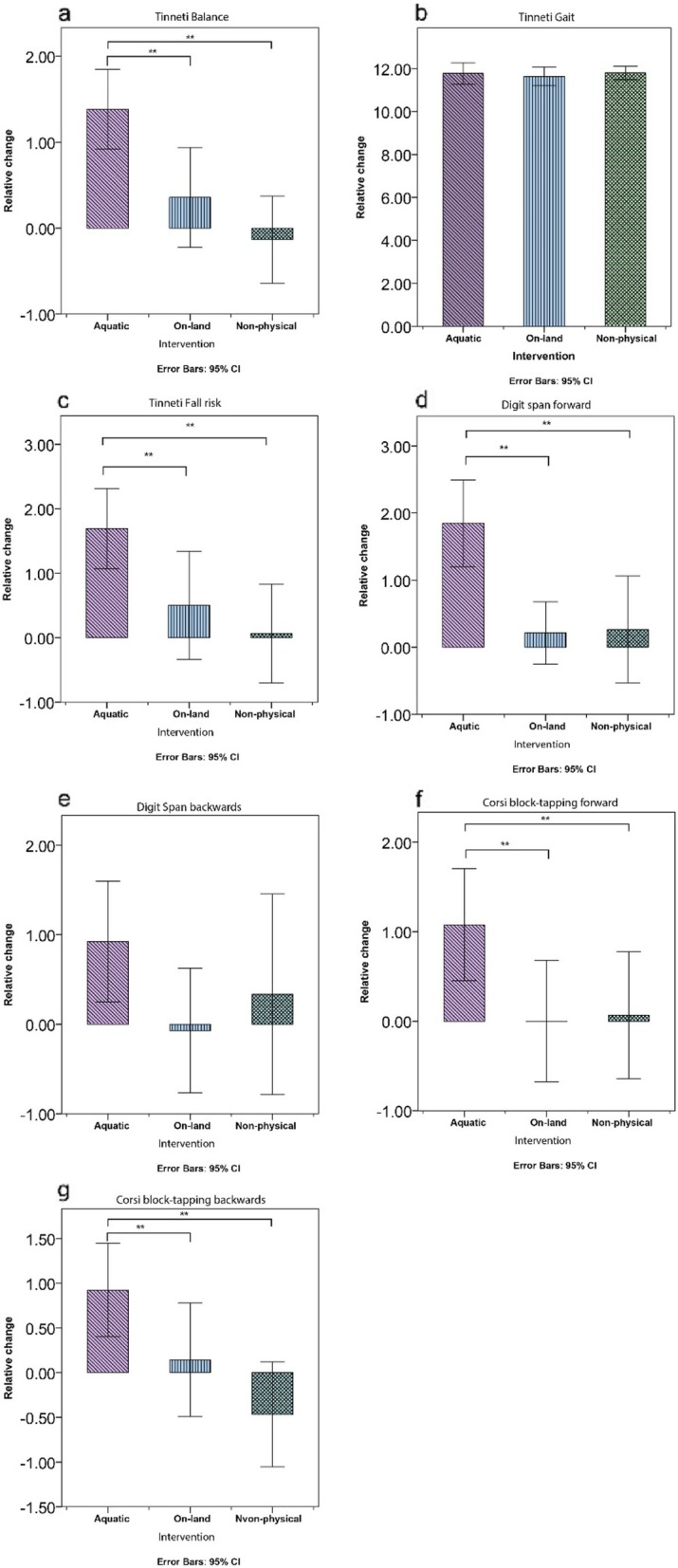
measured parameters after 12 weeks of intervention

The differences in Tinetti balance scores between the baseline measurement and after 12 weeks of intervention between the groups were significant (F(2,39) = 10.03, *p* < 0.001), the API group (M = 1.385, SD = 0.76), the OLPI group (M = 0.357, SD = 1) and the NPI group (M = -0.133, SD = 0.91). Tukey Post Hoc tests showed that both OLPI group and NPI groups improved significantly less than the API group (Fig. 2.a).

**Fig. 3:**
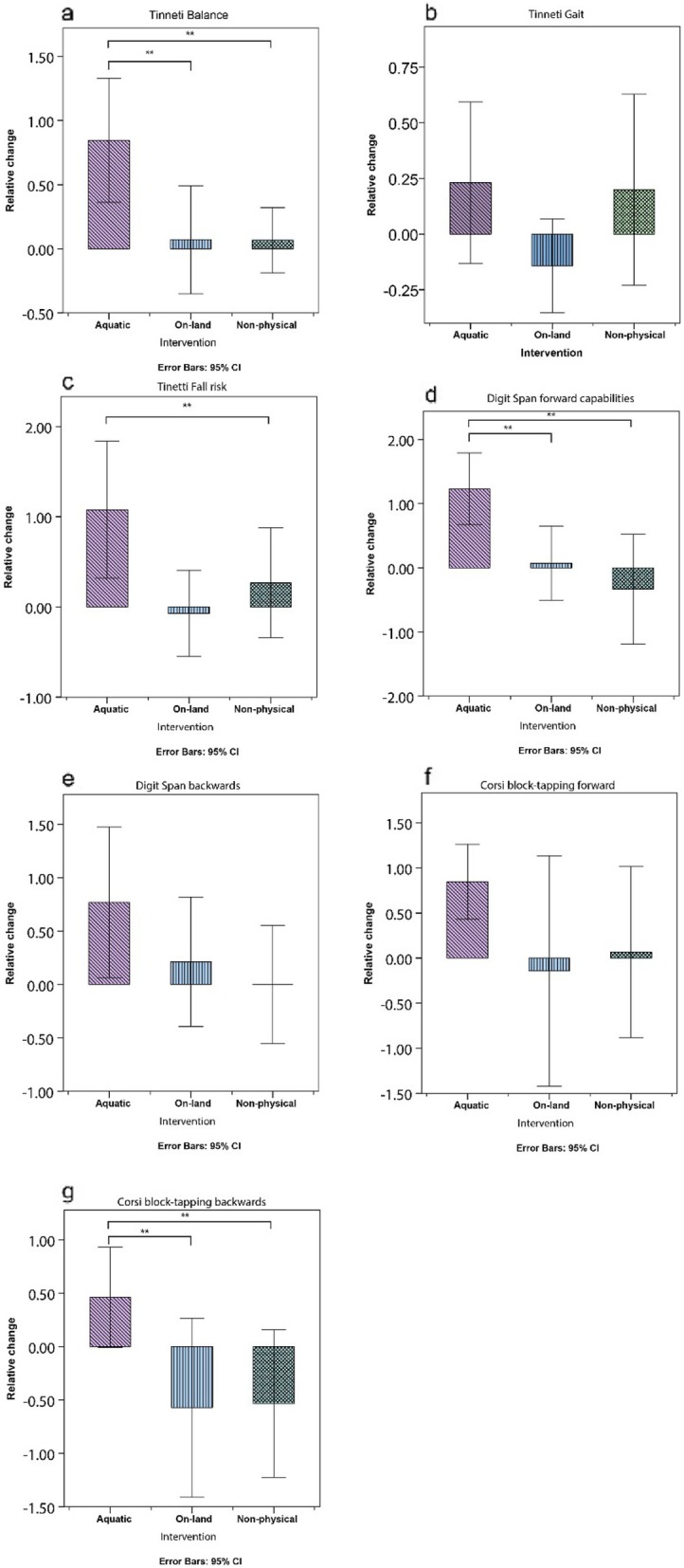
measured parameters after 6 weeks of intervention

The differences in Tinetti balance scores between the baseline measurement and after six weeks of intervention between the groups were significant (F (2, 38)=6.00, *p* < 0.05), the API group (M = 0.846, SD = 0.80), the OLPI group (M = 0.071, SD = 1), and the NPI group (M = 0.66, SD = 0.74). Tukey Post hoc tests showed that both OLPI and NPI groups scored significantly lower than the API group (Fig. 3.a).

The differences in Tinetti gait scores between the baseline measurement and after 12 weeks of intervention between the groups were not significant (F (2, 38)=0.52, n.s) (Fig. 2.b). The differences in Tinetti gait scores between the baseline measurement and after six weeks of intervention between the groups were not significant (F (2, 38)=1.61, n.s) (Fig. 3.b).

The differences in fall risk scores between the baseline measurement and after 12 weeks of intervention between the groups were significant (F (2, 38)=5.62, p0 > .05), the API group (M = 1.69, SD = 1.03), the OLPI group (M = 0.50, SD = 1.45), and the NPI group (M = 0.06, SD =1.38). Tukey Post hoc tests showed that the NPI group scored significantly lower than the API group. The OLPI and NPI groups did not differ significantly (Fig. 2.c).

The differences in Fall risk scores between the baseline measurement and after six weeks of intervention between the groups were significant (F (2, 38)=4.06, *p* < 0.05), the API group M = 1.07, SD = 1.25), the OLPI group (M = 0.26, SD = 1.09), and the NPI group (M = -0.07, SD = 0.82). Tukey Post hoc tests showed that the NPI group scored significantly lower than the API group, and the OLPI group did not score significantly lower than the API group. However, the OLPI and NPI groups did not differ significantly (Fig. 3.c).

The differences in DSF scores between the baseline measurement and after 12 weeks of intervention between the groups were significant (F (2, 38)=8.85, *p* < 0.001), the API group (M = 0.214, SD = 0.80), the OLPI group (M = 1.84, SD = 1.06), and the NPI group (M = 0.26, SD = 1.34). Tukey Post hoc tests showed that both OLPI group and NPI groups scored significantly lower than the API group. However, the OLPI and NPI groups did not differ significantly (Fig. 2.d).

The differences in DSF scores between the baseline measurement and after six weeks of intervention between the groups were significant (F (2, 38)= 6.20, *p* < 0.05), the API group (M = 1.23, SD = 0.92), the OLPI group (M = 0.07, SD = 0.99), and the NPI group (M = -0.33, SD = 1.54). Tukey Post hoc tests showed that the OLPI and NPI groups scored significantly lower than the API group. However, the OLPI group and NPI groups did not differ significantly (Fig. 3.d).

The differences in DSB scores between the baseline measurement and after 12 weeks of intervention between the groups were not significant (F (2, 38)=1.437, n.s), the API group (M = 0.92, SD = 1.11), the OLPI group (M = -0.07, SD = 1.2), and the NPI group (M = 0.33, SD = 2.02) (Fig. 2.e).

The differences in DSB scores between the baseline measurement and after six weeks of intervention between the groups were not significant (F (2, 38)=1.882, n.s), the API group (M = 0.769, SD = 1.16), the OLPI group (M = 0.21, SD = 1.05), and the NPI group (M = 0.00, SD = 1.00) (Fig. 3.e).

The differences in CBTF scores between the baseline measurement and after 12 weeks of intervention between the groups were significant (F (2, 38)=3.54, *p* < 0.05), the API group (M = 1.07, SD = 1.03), the OLPI group (M = 0.000, SD = 1.17), and the NPI group (M = 0.66, SD = 1.27). Tukey post hoc tests showed that both the OLPI and NPI groups scored significantly lower than the API group. However, the OLPI and NPI groups did not differ significantly (Fig. 2.f).

The differences in CBTF scores between the baseline measurement and after six weeks of intervention between the groups were not significant (Fig. 3.f).

The differences in CBTB scores between the baseline measurement and after 12 weeks of intervention between the groups were significant (F (2, 38)=6.50, p < 0.05), the API group (M = 0.923, SD = 0.862), the OLPI group (M = 0.142, SD = 1.09), and the NPI group (M = -0.467, SD = 1.06). Tukey Post Hoc tests showed that the API group scored significantly higher than the NPI group but not from the OLPI. However, the OLPI and NPI groups did not differ significantly (Fig. 2.g).

The differences in CBTB scores between the baseline measurement and after six weeks of intervention between the groups were marginally significant (F (2, 38)=31.9, *p* = 0.052), the API group (M = 0.461, SD = 0.77), the OLPI group (M = -0.57, SD = 1.45), and the NPI group (M = -0.53, SD = 1.24). Tukey post hoc tests showed that both the OLPI and NPI groups scored marginally significantly lower than the API group. However, the OLPI and NPI groups did not differ significantly (Fig. 3.g).

### Secondary outcomes measures

**Fig. 4:**
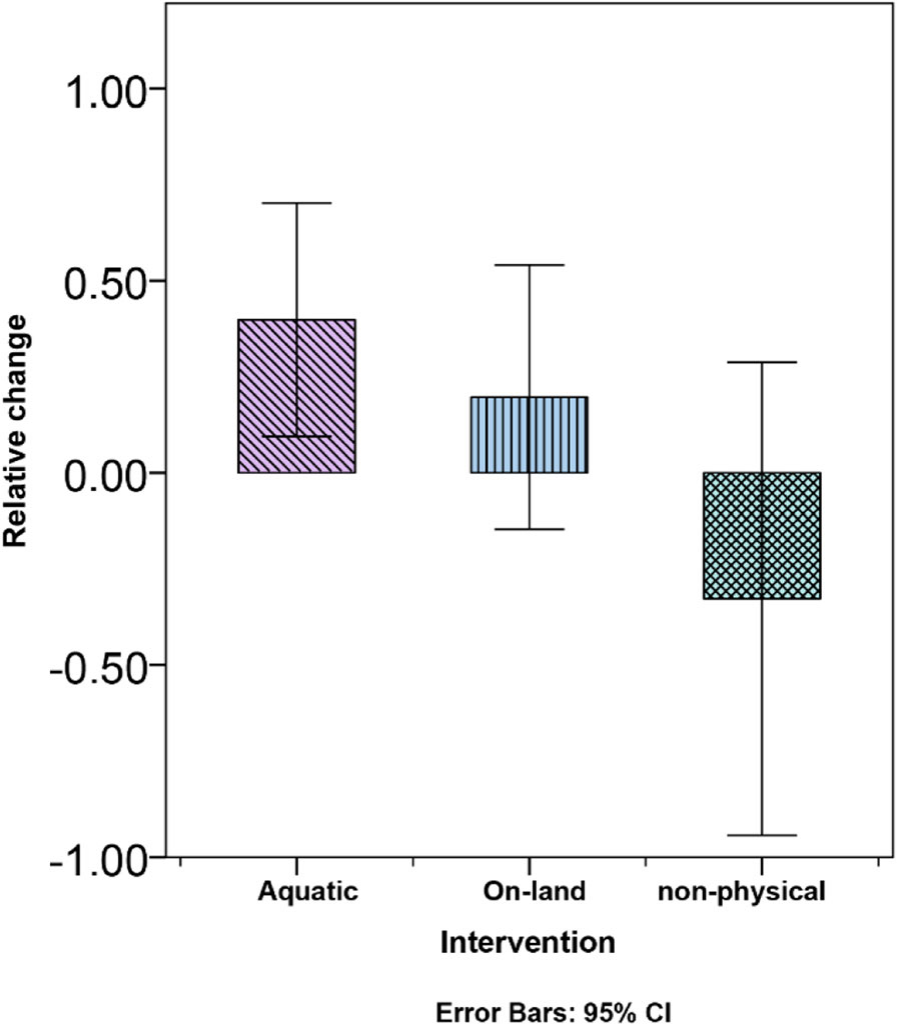
Hazard Perception Test for Pedestrians after 12 weeks of intervention, no significant difference was found between group

The differences in HPTP scores between the baseline measurement and after 12 weeks of intervention between the groups were marginally significant (F (2, 38)=3.13, *p* = 0.055), the API group (M = 0.39, SD = 0.50), the OLPI group (M = 0.19, SD = 0.59), and the NPI group (M = -0.32, SD = 1.11). Tukey post hoc tests showed that the difference between the API group (M = 0.39, SD = 0.50) and the NPI group (M = -0.32, SD =1.11) are marginally significant. The other contrasts did not show significant differences between groups (Fig. 4).

## Discussion

The first aim of the present study was to assess whether API, OLPI or NPI induced different effects on balance, gait and fall risk in older adults.

The study found that after six and 12 weeks of intervention, the API group achieved higher improvement on fall risk score as compared to the NPI group. Previous studies have found that the effect of OLPI, such as Tai-Chi, on fall risk was promising but inconclusive [49–52]. The current study results are in accordance with previous publication [32] suggest that the Ai-Chi method can help to reduce fall risk. However, caution is advised before generalizing the results since this is a pilot study with a small sample size.

As poor balance function is a risk factor for falls, previous studies have found that Tai-Chi practice can improve balance function [49]. However, this study found that after six and 12 weeks of intervention, both OLPI and NPI groups achieved less improvement on balance compared to the API group. These results are in line with previous study that found improvement in both static and dynamic balance in older people after 12 weeks of Ai-Chi program [41]. However, there was no additional intervention for the control group of this study. A possible explanation for the difference in results between OLPI to API may be the environmental uniqueness of the aquatic environment that provides additional resistance for developing balance reactions. The increased multisensory stimulation, induced by the viscosity and the turbulence of water [53], along with the extended time given for balance correction in water, help increase the degree of response variability required for movement control during unstable conditions [54]. Thus, practices in water may assist balance control on-land in spite of the different environment.

In regards to gait, no differences were found between the intervention groups after six or 12 weeks of intervention. However, more than 80% of all participants scored the best possible score of this measure at baseline. Therefore, a ceiling effect was believed to have occurred, and future studies should consider a more sensitive tool for measuring gait among elderly.

Interestingly, both balance and fall risk scores improved after six weeks of intervention among the API group as compared to the two intervention groups. These results suggest that the Ai-Chi method has a faster and better effect on balance and fall risk. Therefore, in cases of limited time or budget, the Ai-Chi method is recommended as a more economic intervention for fall prevention.

The second goal of the present study was to assess whether API, OLPI or NPI induced differential effects on verbal and visuospatial WM in older adults. The current study found that after 12 weeks of intervention, both OLPI and NPI group scored significantly lower than the API group on the DSF test (verbal WM) and on CBTF test (visuospatial WM). No difference was found between the intervention groups in the DSB test and only the NPI group scored significantly lower than the API group after 12 weeks of intervention on the CBTB test. The forward span assesses the ability to maintain verbal information for a brief period of time (remembering digits sequence) while the backward span requires both maintenance of information and manipulation of the items (remembering and reversing the digits sequence) [55]. Thus, it can be argued that the backward span is a more complicated task.

WM performances are affected by age, especially in tasks requiring visuospatial information processing [56]. Previous studies among older adults showed positive effects of physical intervention on WM [19, 20]. However, most of these studies report a positive relationship between aerobic exercise and improve memory function [57, 58]. Tai-Chi is considered to be a low-intensity exercise [59, 60]. Ai-Chi is performed in warm water and has the advantages of the aquatic environment. In warm water, the heart rate rises, contributing to a further rise in cardiac-output [28]. Previous studies have found that water immersion increases both the rate and volume of blood-flow to the brain [61, 62]. Thus, the increase in WM, as measured by the DSF, CBTF and CBTB tests, may be caused by the effects of water immersion, leading to improved brain function.

The third goal of the present study was to assess whether aquatic physical, on-land physical or non-physical interventions induced differential effects on hazard-perception in older adults. The current study found a trend of improvement on the HPTP score after 12 weeks of intervention only among the API group. Safe walking and road-crossing demand cognitive and motor skills such as the allocation of attention resources, estimation of speed and distance, WM and coordination of numerous sub-tasks [63, 64]. Additionally, safe road-crossing requires a hazard-perception which enables anticipation of dangerous situations on the road ahead [65, 66]. Strengthening motor, cognitive and hazard-perception abilities demonstrates a bottom-up approach to safe road-crossing, an essential skill required for participation in every-day life [67, 68].

This study should be viewed in light of its limitations. Specifically, given its small sample size, caution is advised before generalizing the results. In addition, 16.66% of participants were not randomly allocated. Moreover, since an aquatic NPI group was not included in this study, it is unclear whether the outcomes are related to either the activity within the aquatic environment or to the immersion itself. Finally, while the current study examined the effects of activities after 12 weeks, no long-term effects were examined. Thus, future studies should test long term effects.

## Conclusions

This study demonstrated the benefits of API for decreasing fall risk, while improving balance, verbal and visuospacial WM. In addition, a trend of improvement in hazard-perception was found. While the results indicate that API may help promote safe road-crossing, this method should also be examined in real life situations. Future studies should also test API’s effects on brain activity for better understanding of the neuronal mechanism underline these changes.The findings and conclusions of the research may affect clinicians working with the elderly when making decisions concerning therapeutic prevention interventions for healthy elderly individuals. Such interventions may help reduce falling and cognitive deficits, which in turn, could reduce injuries and fatalities of older pedestrians.

## Data Availability

The datasets used and/or analysed during the current study are available from the corresponding author on reasonable request.

## Abbreviations

API: Aquatic physical intervention
CBTB: Corsi block-tapping backward
CBTF: Corsi block-tapping test forward
DSB: Digit span test backward
DSF: Digit span test forward
HPTP: Hazard-perception test for pedestrians
NPI: Non-physical intervention
OLPI: On-land physical intervention
WM: Working memory
